# Observation of the Primo Vessel Approaching the Axillary Lymph Node with the Fluorescent Dye, DiI

**DOI:** 10.1155/2014/287063

**Published:** 2014-11-16

**Authors:** Su Youn Park, Byung-Soo Chang, Seung Hwan Lee, Ju Hwan Yoon, Sungchul Kim, Kwang-Sup Soh

**Affiliations:** ^1^Nano Primo Research Center, Advanced Institute of Convergence Technology, Seoul National University, Suwon 443-270, Republic of Korea; ^2^Physics Department, Gachon University, Seongam 461-701, Republic of Korea; ^3^Department of Cosmetology, Hanseo University, Seosan 356-706, Republic of Korea; ^4^Department of Acupuncture & Moxibustion, Gwangju Medical Hospital, Wonkwang University, Gwangju 503-310, Republic of Korea

## Abstract

The primo vascular system (PVS) floating in lymph fluid has mostly been observed in large caliber ducts around the caudal vena cava and the thoracic duct of rabbits, rats, and mice. But the PVS has not been traced up to the lymph nodes. It has not been established whether the PVS leaves the lymph vessel through the lymph vessel wall or it enters the lymph nodes. Therefore, observing the PVS entering a lymph node, for example, the axillary node, is desirable. In the current work, we traced the PVS approaching up to the surface of axillary node of a rat. The method used for this study was based upon a method that was recently developed to detect the PVS in the lymph duct from the inguinal to the axillary nodes in the skin of a rat by injecting Alcian blue into the inguinal node. However, the Alcian blue blurred near the lymph nodes and tracing the PVS up to the lymph nodes has not been possible. The current method clearly showed the PVS approaching the axillary node.

## 1. Introduction

Although the primo vascular system (PVS) was originally noticed more than fifty years ago [1, 2], its confirmation was only recently made because no method for its observation was described in the original literature. The PVS had several subsystems, one of which was the one in skin and was claimed to be the anatomical structure corresponding to acupuncture points and meridians [1, 2]. Other subsystems were directly or at least indirectly influenced by acupuncture treatments because they are connected to the acupuncture meridians as parts of the whole circulatory system, PVS according to Kim [1]. Among the other subsystems there was the PVS floating in the fluid of lymph ducts [2]. Recently, this lymph-associated PVS (L-PVS) was studied extensively by using several methods with various dyes: in the case of rabbits, it was studied with the dyes Janus green B [3] and Alcian blue [4, 5] and with optical methods without injecting a dye [6]. In the case of rats, it was studied with magnetic fluorescence nanoparticles [7] and the dye Alcian blue [8], and, in the case of a Prox1-GFP transgenic mouse, it was studied without the injection of dyes [9]. A rare case was a primo vessel in a lymph vessel that came from a tumor that had been xenografted in the ventral skin of a mouse [10]. This case was a reinforcing example of the conjectured role of the PVS in cancer metastasis [11].

Recently, the PVS was observed for the first time in the lymph duct running from the inguinal to the axillary lymph nodes in the skin by injecting Alcian blue into the inguinal lymph node [12]. This was a big step forward in the sense that it had become possible to observe the L-PVS for a long period of time without severe surgery because it will only need a window system to observe the lymph in skin. Such monitoring will lead to a physiological understanding of the periodic generation of cells in primo nodes, as claimed by Kim [2].

However, it was not shown whether the primo vessel came out of the lymph vessel through the lymph wall or continued to enter the lymph node because the Alcian blue blurred near the lymph node and therefore clear tracing was not possible near the lymph nodes. In the current work we found that another dye, 1,1′-dioctadecyl-3,3,3′3′-tetramethylindocarbocyanine perchlorate (DiI), worked better to trace the primo vessel up to the surface of the axillary lymph node. Thus, for the first time, the L-PVS approaching the axillary node was shown. Because, in previous works, the L-PVS had not been traced up to nodes [3–10, 12], the present work is a significant progress toward complete tracing of the L-PVS in the lymph system.

A possible medical significance of the PVS was suggested by the observation of abundant immune cells in the L-PVS [8]. Presence of hyaluronic acids and of the hormones adrenalin and noradrenalin in the PVS, as claimed earlier by Kim et al. [2, 8, 13], was confirmed. In addition, the role of the L-PVS in cancer metastasis through the lymph duct [10, 11] is an important issue.

## 2. Materials and Methods

### 2.1. Preparation of Animals

Ten male Sprague-Dawley (SD) rats (6 weeks old) were obtained from DooYeol Biotech (Seoul, Korea). The animals were housed under constant temperature and humidity conditions (23°C, relative humidity: 60%) with a 12 hour/12 hour light/dark cycle and were provided water and commercial rat chow* ad libitum*. The rats were anesthetized with urethane (1.5 g/kg) and xylazine (20 mg/kg) administered intramuscularly, which allowed all surgical procedures to be performed under systemic anesthesia. The procedures involving the animals and their care were in full compliance with current international laws and policies (*Guide for the Care and Use of Laboratory Animals*, National Academy Press, 1996). The experiment was approved by the Institutional Ethics Committee of the Advanced Institute of Convergence Technology (Approval number: WJIACUC20130212-1-07).

### 2.2. Visualization of the Primo Vascular System with DiI

The DiI powder (Sigma Aldrich, St. Louis, USA) was dissolved in 99.9% ethyl alcohol to make a stock solution that was kept in a refrigerator (4°C) after having been wrapped with aluminum foil to avoid exposure to light. The staining dye was the working solution that was made from the stock solution (10 *μ*L diluted with 1 mL of 70% ethyl alcohol).

An incision in the subcutaneous layer of the skin along the linea alba was performed with surgical scissors, and the incised skin was bent back to expose the target inguinal lymph nodes. The DiI was injected into one of the several inguinal nodes. The injection needle was a glass microcapillary that was fixed to a 1 mL syringe (gauge 26).

In order to help the dyeing agent to be thoroughly washed out from the lymph duct, natural circulation of the lymph fluid had to be maintained as much as possible. One necessary thing was to keep the normal body temperature of rats. For this, covering the rats with paper tissue was a simple but effective means. For two hours after the dye had been injected, the rat was in a resting state in order to allow the dye to be absorbed by the L-PVS and to be washed out by the natural lymph flow. The target lymph duct between the inguinal and the axillary nodes of the rat was exposed to detect the L-PVS in it. All procedures of the observation and the operation were performed under a stereomicroscope (SZX12, Olympus, Japan).

### 2.3. Observation of the Primo Vascular System

In order to detect the L-PVS* in vivo*, we used a fluorescence tissue microscope (MVX10, Olympus, Japan) with an excitation filter for the wavelength *λ* = 530~555 nm and barrier filter with *λ* = 570~625. The maximum absorption and emission wavelength of DiI were 550 nm and 567 nm, respectively. The lymph duct and its L-PVS were photographed, and the images were displayed on a monitor (C240P4QPYEW100, Philips, Japan). The surrounding fat tissues had to be removed with microforceps. Although the lymph duct was orange colored due to the unwashed remnant dye, the L-PVS glowed more strongly, thus revealing its presence.

After the stained L-PVS in the lymph duct had been observed, the rats were sacrificed by intracardiac injection of urethane (1 mL). A piece of the lymph duct including the stained L-PVS was cut with microscissors from the rat under the fluorescence tissue microscope. It was put on a slide glass with 1x PBS and was observed with a phase contrast microscope (BX51TRF, Olympus, Japan). The specimen was fixed with 10% NBF and kept in a refrigerator at 4°C.

For morphological analysis, the primo vessel was extracted from the lymph duct. For the staining of nuclei and f-actins in the cells, 4′,6-diamidino-2-phenylindole (DAPI) and phalloidin were applied, respectively. The specimen was stained with a 300 nM DAPI (D1306, Invitrogen, MO, USA) solution for 20 minutes and a 6.6 *μ*M phalloidin 488 (A12379, Invitrogen, MO, USA) solution for 30 minutes. After the specimen had been washed, it was covered with mounting solution. The stained specimen was investigated under a phase contrast microscope (BX51TRF, Olympus, Japan).

## 3. Results

The basic morphological data of the lymph vessels and the primo vessels observed in the current work with DiI are summarized in Table 1. The diameters of the lymph and the primo vessels were 155.4 ± 103.7 *μ*m and 41.4 ± 31.5 *μ*m, respectively. These data are consistent with previous reports on lymphatic primo vessels [12].

**Figure 1:**
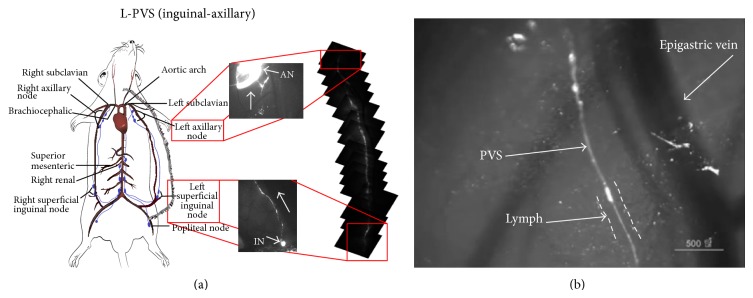
Visualization of the primo vascular system in the skin lymph duct between the inguinal and the axillary nodes with the fluorescent dye DiI. (a) A schematic diagram of large-caliber lymph ducts of a rat. The lymph duct between the two nodes is near the epigastric blood vessel, which is the largest and easily identifiable vessel in this area of the skin. The axillary lymph node (AN; middle upper panel) became bright due to the DiI that flowed in through the lymph duct from the inguinal node (IN; middle lower panel). The DiI was injected into the inguinal node, and it flowed up to the axillary node through the lymph duct. The arrows in the middle two panels indicate the flow direction of DiI. Mosaics of the fluorescent images of the primo vessel in the lymph duct connecting the two nodes are given in the right panel. (b) Magnified view: A primo vessel stained by DiI was clearly visible in the lymph duct whose boundary was hardly visible. The PVS-containing lymph duct was just beside a small vessel that ran parallel to the epigastric blood vessel. This image demonstrates that DiI was not strongly absorbed by the lymph wall.

The PVS was observed in the skin lymph duct along the epigastric vessel from the inguinal to the axillary nodes (Figure 1(a)). The inguinal node was bright due to the injected DiI, as was the efferent lymph duct due to the flowing DiI. The axillary node was also bright due to the DiI that arrived via the lymph duct, which was barely seen in this figure. The mosaic of the images capturing the entire lymph ducts showed the primo vessel (the right panel of Figure 1(a)). One part was magnified to demonstrate that the primo vessel was clearly visible, but the lymph duct running parallel to the epigastric blood vessels was hardly visible because it was not stained by the DiI (Figure 1(b)).

**Figure 2:**
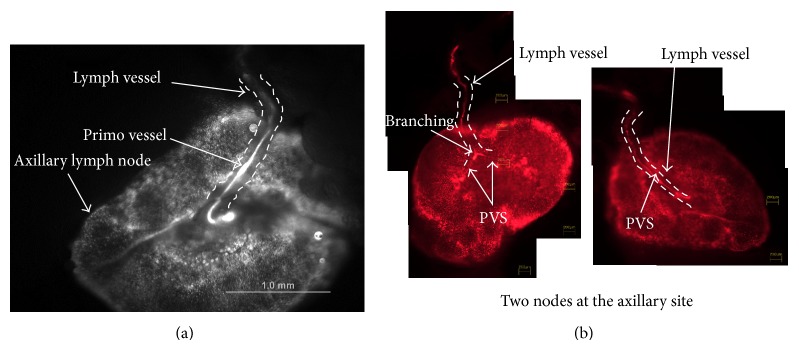
A primo vessel entering the axillary node. (a) A lymph duct entered the axillary lymph node, as did a primo vessel. Notice the white curves of the PVS inside the lymph duct. Black and white stereomicroscope image (MVX10). The scale bar is 1.0 mm. (b) The same node (right-hand side) and its associated node (left-hand side) were seen under a phase contrast microscope. The red color was the DiI fluorescence. The lymph duct branched after meeting the node, and the two branches merged to the capsule of the node. The PVS also branched following the lymph branches.

Figure 2 shows the axillary lymph node into which a lymph duct entered. The lymph duct merged to the membrane of the node. In Figure 2(a), the white curves show the PVS inside the lymph duct. The PVS was white because the stereomicroscope was equipped with a b/w CCD camera (MVX 10, Olympus), which captured the DiI's fluorescence. The PVS also branched following the lymph branches. The same node (the right-hand side of Figure 2(b)) and its associated node (the left-hand side) were observed under a phase contrast microscope. The red color is DiI's fluorescence. This node on the left-hand side showed that the PVS branched, following the branching of the lymph duct, before entering the lymph node.

**Figure 3:**
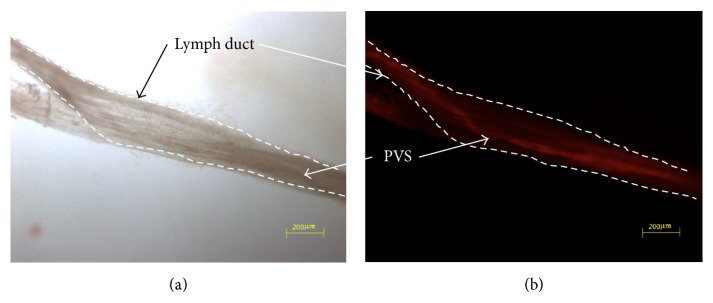
A primo vessel in a lymph duct. (a) A piece of the lymph duct (broken line) was taken, fixed, and put on a slide glass. The primo vessel (arrow) was hardly identifiable. (b) The primo vessel (arrow) showed strong fluorescence due to the DiI. The lymph duct did not show fluorescence, which demonstrated that DiI was absorbed very weakly by the lymph duct.

In the previous work [12], the image was taken with a stereomicroscope, but, in the current work, the primo vessel was only observed with a fluorescent microscope. An isolated lymph duct was fixed with NBF, put on a glass slide, and observed with a phase contrast microscope without fluorescence (Figure 3(a)). The primo vessel was recognizable but could not be clearly identified. The same sample was observed with the same microscope, but with a fluorescence filter, and the primo vessel emerged clearly (Figure 3(b)). This figure also showed that the lymph wall had a very weak fluorescence.

**Figure 4:**
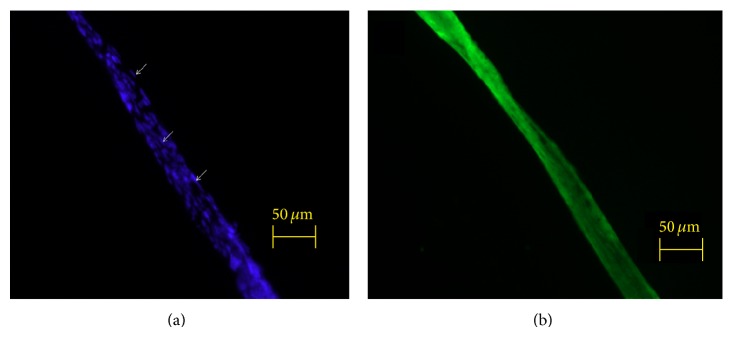
The primo vessel extracted from the lymph duct. (a) DAPI signal image of the primo vessel that was extracted from the lymph duct. Notice some rod-shaped nuclei (arrows), which were similar to those used for the characteristic identification of primo vessels in a previous work [12]. (b) Phalloidin signal image of the primo vessel. The f-actins were along, not transverse to, the vessel. The pattern of the f-actins was different from the patterns of lymphocytes, as was shown in previous work [12].

Figure 4(a) shows that the rod-shaped nuclei stained with DAPI were aligned longitudinally parallel to the primo vessel. The lengths of the nuclei were between 10 and 20 *μ*m, as expected [1]. The phalloidin staining signals of the f-actins in the cells (Figure 4(b)) were also aligned along the direction of the vessel.

## 4. Discussion

The diameters of the primo vessels that were observed in blood vessels, on organ surfaces, and in brain ventricles were around 20–30 *μ*m. Sometimes the observed diameters were much larger than the normal values, which might have been caused by the other debris that was attached to the primo vessels, as shown in the Alcian blue staining cases [12].

We examined the samples for identifying a primo vessel. The hallmark of a primo vessel is the distribution, as well as the shapes and lengths, of nuclei. Another hallmark is the pattern of the f-actins. These two features were in good agreement with those reported in previous PVS works [12].

The PVS in the lymph ducts from the inguinal to the axillary nodes was first found and identified in a previous work with the aid of Alcian blue [12]. But the detailed approach to the lymph nodes was not studied because the Alcian blue was all blurred near the surfaces of lymph node. In the current work, another dye, DiI, was used which clearly showed the primo vessel approaching the surface of the axillary node. Thus, we established that the primo vessel did not come out of the lymph vessel through the lymph wall but entered the lymph node. This work will be helpful to further trace the PVS into the lymph node which requires another technique because the current and previous staining methods cannot be used inside a lymph node. Since we got precise information through this experiment on the point where the primo vessel enters the lymph node we can apply the histological investigation on this part of a lymph node.

About the possible relevance with respect to acupuncture, an L-PVS was shown to be connected to the organ-surface PVS which was distributed on the surfaces of internal organs [6]. On the other hand, to the organ-surface PVS flowed the Alcian blue that was injected into the acupuncture point BL-23 of a rat [14]. Therefore, the connection of the L-PVS to the acupuncture point was shown by combination of the two experiments. More experiments showing the flow of primo fluid from acupuncture points to the L-PVS are highly desirable.

## Supplementary Material

It is a movie which was recorded by a fluorescent microscope, MVX10(Olympus, Japan). The video shows movement of the primo vessel (white thin line) which is visible due to the fluoresence of the dye DiI. The lymph duct is milky white line surrounding the primo vessel. The dark thick line to the right of the lymph duct is the epigastric blood vessel.

## Figures and Tables

**Table 1:** Diameters of the primo vessels and the lymph ducts between the inguinal and the axillary lymph nodes of rats. The animals were all 6-week-old, male, Sprague-Dawley rats.

Rat identification number	Diameter of lymph duct (*μ*m)	Diameter of primo vessel (*μ*m)
1	144.0	42.6
2	134.4	21.6
3	253.8	88.2
4	380.1	91.3
5	221.3	67.8
6	108.4	45.6
7	140.4	24.3
8	62.0	18.5
9	66.6	8.4
10	43.1	6.2

Average ± SD	155.4 ± 103.7	41.4 ± 31.5

## References

[1] Kim B. H. (1963). On the Kyungrak system. *Journal of the Academy of Medical Sciences of the Democratic People's Republic of Korea*.

[2] Kim B. H. The Kyungrak system. *Proceedings of the Academy of Kyungrak of the DPRK*.

[3] Lee B.-C., Yoo J. S., Baik K. Y., Kim K. W., Soh K.-S. (2005). Novel threadlike structures (Bonghan ducts) inside lymphatic vessels of rabbits visualized with a Janus Green B staining method. *The Anatomical Record Part B: The New Anatomist*.

[4] Lee C., Seol S.-K., Lee B.-C., Hong Y.-K., Je J.-H., Soh K.-S. (2006). Alcian blue staining method to visualize Bonghan threads inside large caliber lymphatic vessels and X-ray microtomography to reveal their microchannels. *Lymphatic Research and Biology*.

[5] Noh Y.-I., Rho M., Yoo Y.-M., Jung S. J., Lee S.-S. (2012). Isolation and morphological features of primo vessels in rabbit lymph vessels. *Journal of Acupuncture and Meridian Studies*.

[6] Lee B.-C., Soh K.-S. (2008). Contrast-enhancing optical method to observe a Bonghan duct floating inside a lymph vessel of a rabbit. *Lymphology*.

[7] Johng H.-M., Yoo J. S., Yoon T.-J., Shin H.-S., Lee B.-C., Lee C., Lee J.-K., Soh K.-S. (2007). Use of magnetic nanoparticles to visualize threadlike structures inside lymphatic vessels of rats. *Evidence-Based Complementary and Alternative Medicine*.

[8] Kwon B. S., Ha C. M., Yu S., Lee B. C., Ro J. Y., Hwang S. (2012). Microscopic nodes and ducts inside lymphatics and on the surface of internal organs are rich in granulocytes and secretory granules. *Cytokine*.

[9] Choi I. H., Chung H. K., Hong Y. K., Soh K. S., Kang K. A., Harrison D. (2011). Detection of the primo vessels in the rodent thoracic lymphatic ducts. *The Primo Vascular System*.

[10] Lee S. W., Ryu Y. H., Cha J. M., Lee J.-K., Soh K.-S., Kim S. C., Lim J. K. (2012). Primo vessel inside a lymph vessel emerging from a cancer tissue. *JAMS Journal of Acupuncture and Meridian Studies*.

[11] Yoo J. J. S., Kim H. B., Won N. (2011). Evidence for an additional metastatic route: *in vivo* imaging of cancer cells in the primo-vascular system around tumors and organs. *Molecular Imaging and Biology*.

[12] Lee S. H., Bae K.-H., Kim G. O. (2013). Primo vascular system in the lymph vessel from the inguinal to the axillary nodes. *Evidence-based Complementary and Alternative Medicine*.

[13] Kim J. D., Ogay V., Lee B. C., Kim M. S., Lim I., Woo H. J., Park H. (2008). Catecholamine producing novel endocrine organ: bonghan system. *Medical Acupuncture*.

[14] Han H.-J., Sung B., Ogay V., Soh K.-S. (2009). The flow path of alcian blue from the acupoint BL23 to the surface of abdominal organs. *JAMS Journal of Acupuncture and Meridian Studies*.

